# Linear Scar Face Re-orientation vs Resurfacing: Reviving old paradigm

**DOI:** 10.1016/j.jpra.2025.02.014

**Published:** 2025-03-06

**Authors:** Ahmed Sobhi Hweidi, Shahd Mahdy, Paul McArthur

**Affiliations:** 1 Assistant Professor of Plastic, Burn and Maxillofacial Surgery Department at Ain Shams University Hospitals, Cairo, Egypt; 2 Plastic Surgery Resident at Plastic, Burn and Maxillofacial Surgery Department at Ain Shams University Hospitals, Cairo, Egypt; 3 Consultant Plastic & Reconstructive Surgery, Mersey Regional Plastic Surgery unit Liverpool, UK

**Keywords:** fractional laser resurfacing, Z-plasty, facial scars, scar revision, patient satisfaction

## Abstract

The study highlights the comparative effectiveness of surgical scar re-orientation combined with fractional laser resurfacing versus isolated laser treatment for linear facial scars. The results demonstrated that patients who underwent the combined approach achieved superior outcomes in terms of scar appearance and psychological satisfaction. This study can encourage the re-evaluation of addressing scars and further emphasizes the need for treatment plans that combine surgical and non-surgical modalities to optimize patient outcomes and their quality of life. Future studies with larger sample sizes and randomized controlled trials are recommended to further validate these findings.

## Introduction

The development of fractional laser technology introduced a minimally invasive approach to skin resurfacing, which has gained popularity since its emergence in the early 1990s. Multiple studies have demonstrated that fractional laser treatments stimulate collagen production and promote skin renewal, making them valuable in scar management.^1^^,^^2^ Although it has been successful in enhancing scar appearance, it is not known if isolated laser resurfacing is sufficient to significantly improve linear facial scars.

We searched for an answer by revisiting previously used surgical modalities in scar revision, one of which is linear scar re-orientation, which can be achieved by z-plasty, w-plasty or geometric line closure. Notably, these techniques are not new concepts and were mainly used for reconstruction; therefore, they are functional rather than being used for cosmetic purposes. Morestin in 1914 described the technique used to treat retracted scars of the hand.^3^ Later on in 1952, Hazarti adapted this technique on depressed scars by reorienting them without interfering with the relaxed skin tension lines.^4^ McGregor in 1966 succeeded in being known as one of the prominent advocates for this technique; he highlighted its importance in multiple reconstructive purposes and researched in depth its cosmetic value in facial scars. He emphasized the camouflage effect on linear scars when they are converted into multiple small zigzag ones.^5^ Despite the proven track record, focus was diverted towards the evolving minimally invasive methods for scar revision. In our experience this it was challenging to convince patients, in our practice to undergo surgical re-orientation with several patients believing that laser treatment alone would provide better outcomes.

Past experiences of positive scar re-orientation, using z-plasty encouraged the team to consider a new method of treatment. Although we were aware that laser CO_2_ resurfacing could improve the appearance of a scar, we investigated if surgical re-orientation before performing additional laser resurfacing can improve the initial scar.

A prospective study based on previous outcomes was devised to compare 2 treatment groups, those with laser CO_2_ resurfacing in isolation against a group who underwent scar re-orientation and adjuvant laser treatment.

## Materials and Methods

This prospective, comparative study included 2 groups of patients:

Group A: involved 63 patients who chose to undergo isolated laser resurfacing performed using AMI BX 300 (MESODERMA) machine.
Group B: Included 59 patients who underwent surgical revision via multiple z-plasties followed by adjuvant laser treatment.
Group allocation was through patient choice after thorough pre-treatment consultation.

Patients were selected based on their scar location, predominantly facial, and orientation that mainly included linear appearance. Most patients experienced emotional distress due to the scar.

Patients were evaluated by clinical history and examination of the scar; this was mainly to exclude contraindications to surgical revision such as autoimmune connective tissue disorders or psychiatric illness that would hinder realistic expectations. The scar assessment was based on preoperative and post-operative Manchester scar scale which is used to assess the scar color, texture, distortion and contour with an added visual analogue scale (VAS) that was rated by the lead surgeon.^6^

Although it was not a very popular score, it has been used during scar assessment for 10 years in our practice and we believe that it would add great value and reliability to the evaluation of scars in this study.

Post-operative satisfaction was also measured using VAS rated by the patient.

Regarding group A, 63 patients opted for minimally invasive resurfacing using fractional CO_2_ laser. Patients underwent an average of 6 to 8 sessions that was scheduled every month. Thirty minutes prior to each session, a topical local anesthetic cream was applied.

In contrast, patients in group B, who underwent surgical revision, comprised 59 patients who were prepared by signing an informed consent explaining the procedure. This was followed by skin marking of the z-plasty design and photographic documentation.

## Surgical technique

Local anesthetic preparation with epinephrine was injected with a total waiting time of 15 min prior to incision.

This was followed by sterilization and toweling. Incision was made over the preplanned marking. The scar was removed first by a regular ellipse, then the limbs of the z-plasty were incised and dissected thoroughly in a plane superficial to the superficial musculoaponeurotic system. Adequate haemostasis was then ensured, and the limbs were reorientated with subcutaneous suturing by 4-0 Monocryl sutures. Finally, skin closure was performed using permanent prolene 6-0 sutures with compressive dressing on top to obliterate any dead space.

Sutures were removed on the seventh day post-procedure. The first fractional laser session was carried out 2 weeks post-operatively. Laser sessions were continued for 2–4 sessions every month. Our insight regarding fewer sessions of resurfacing is that the scar would be broken into smaller geometric parts, allowing better results.

Post-operative evaluation included assessment of the scar using the Manchester scar scale scoring system and VAS as rated by the lead surgeon. The score was then calculated as a sum of both scales for which higher scores corresponded to poorer scars. Patient satisfaction was also measured using a VAS, where they rated their scar on a scale of 1 to 10, where 1 indicated a perfect scar and 10 a poor one. Outcome was measured according to scoring systems and patients’ satisfaction.

Patients in both groups were subjected to an ongoing assessment for 1 year and were recalled at 3 months and 6 months after their procedures.

## Results

Our study population was divided into 2 groups according to the intervention chosen by the patients; group A included patients who underwent fractional CO_2_ laser resurfacing and comprised 52 men and 11 women. In group B, the patients chose to revise their scars surgically and this group included a total of 59 patients comprising 52 men and 7 women (Table 1). We worked on scars ranging in average length from 9.5 to 9.8 cm in both groups. The scars were mostly found over the cheek, followed by nape scars, then almost equally between the rest of the facial areas such as forehead, nasolabial fold and over the lower face/mandibular angle.

**Table 1:** Demographic and scar characteristics between the study groups

Variables		Z-plasty plus laser group (Total=59)	Laser group (Total=63)	p-value
**Age (years)**	30.6±6.9	32.2±6.7	^0.200	
**Sex**	**Male**	52 (88.1%)	52 (82.5%)	#0.384
	**Female**	7 (11.1%)	11 (17.5%)	
**Length of scar (cm)**	9.8±2.1	9.5±1.9	^0.518	
**Site of scar**	**Cheek**	21 (35.6%)	18 (28.6%)	§0.875
	**Forehead**	8 (13.6%)	11 (17.5%)	
	**Angle of mandible and neck**	9 (15.3%)	12 (19.0%)	
	**Nasolabial fold**	8 (13.6%)	10 (15.9%)	
	**Nape**	13 (22.0%)	12 (19.0%)	

Data presented as Mean±SD or number (%). ^Independent t-test. #Chi squared test. §Fisher's Exact test.

Notably, we have worked with patients who are considered Fitzpatrick skin type II–V, with most of them falling into types III and IV (Table 2). We have observed that there were no huge discrepancies in the results between different skin types with appearance marked satisfactory in terms of scar scores and VAS. All patients underwent surgery after a minimum waiting time of 3 months after the injury is sustained to allow the scar to settle and mature.

**Table 2:** Demographic and scar characteristics between the study groups

Variables		Z-plasty plus laser group (Total=59)	Laser group (Total=63)	p-value
**Age (years)**	30.6±6.8	32.2±6.7	^0.200	
**Sex**	**Male**	52 (88.1%)	52 (82.5%)	#0.384
	**Female**	7 (11.1%)	11 (17.5%)	
**Mode of trauma**	**Sharp object**	44 (74.6%)	42 (66.7%)	#0.583
	**Road traffic accident**	9 (15.3%)	14 (22.2%)	
	**Others**	6 (10.2%)	7 (11.1%)	
**Fitzpatrik type**	**Type II**	4 (6.8%)	8 (12.7%)	#0.359
	**Type II**	18 (30.5%)	17 (27.0%)	
	**Type IV**	30 (50.8%)	35 (55.6%)	
	**Type V**	7 (11.9%)	3 (4.8%)	
**Time from injury (months)**	15.9±5.6	16.7±5.9	^0.419	
**Length of scar (cm)**	9.8±2.1	9.5±1.9	^0.518	

Data presented as Mean±SD. ^Independent t-test. #Chi squared test.

**Figure 1:**
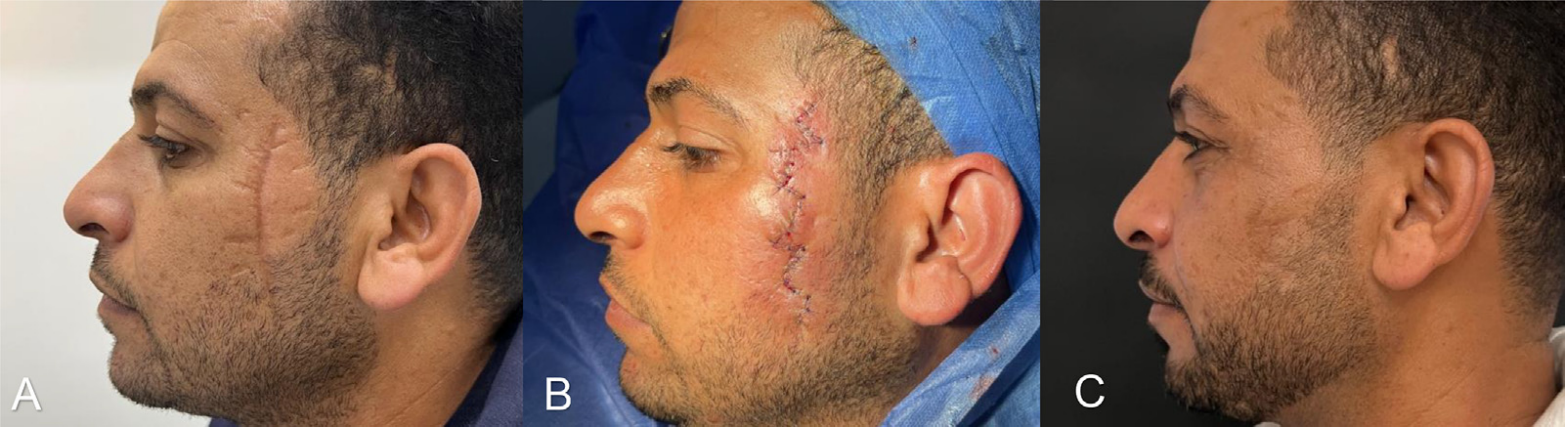
Preoperative photo of the patient's scar on his first visit (A), a railway scar was noticed and was considered during our excision with integration within the z-plasty limbs (B). The patient's scar after 1 year of follow-up (C).

**Figure 2:**
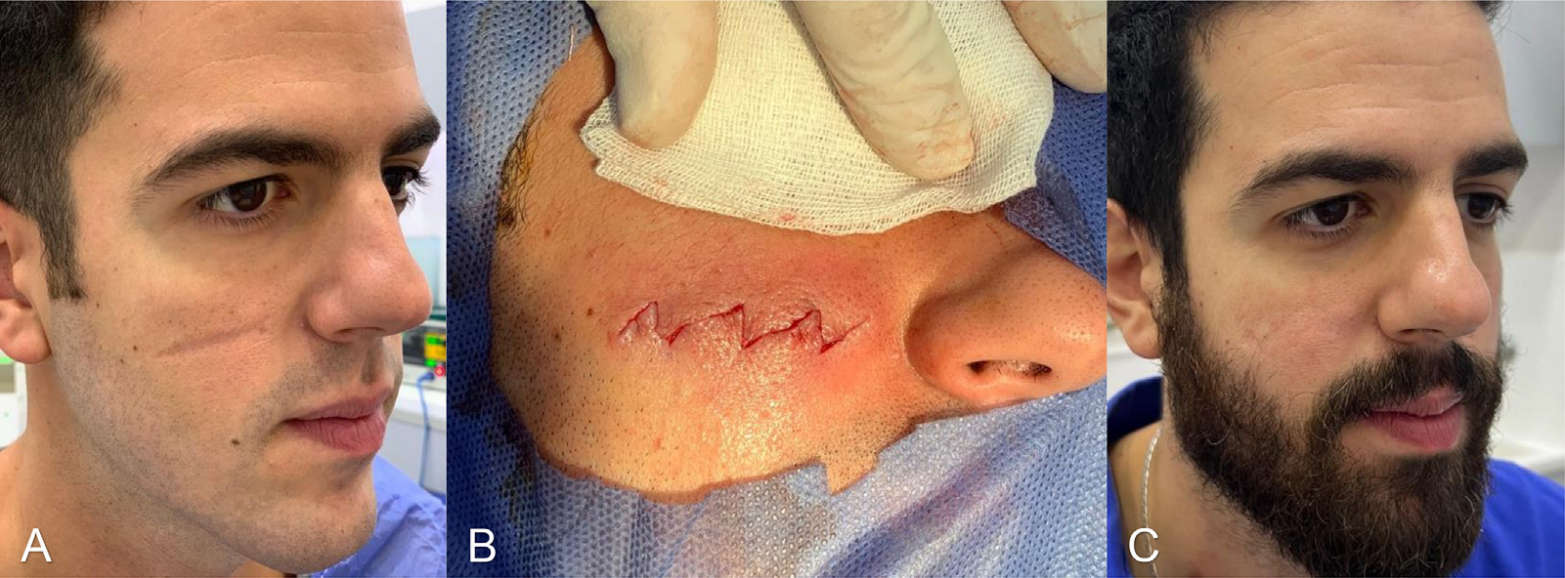
Preoperative photo of the patient's scar on his first visit (A); results after re-orientation of the scar into a zig-zag shape. The patients scar after 1 year of follow-up.

During our follow-up period, significant improvement was observed in both groups of patients between the preoperative and post-operative Manchester scar scores and VAS; however, there was a considerable significant difference in the group B results. Patient satisfaction was also measured subjectively using a VAS from the patients’ point of view. Considerably greater satisfaction was observed in patients who underwent scar re-orientation (Table 3), thus validating our hypothesis. Patients were followed up for 12 months and their pre- and post-operative scar appearance, where photographically documented, are as shown in (Figures 1-2).

**Table 3:** Manchester scar score and visual analogue score domains between the study groups

Variables	Time	Z-plasty plus laser group (Total=59)	Laser group (Total=63)	^p-value
**Manchester scar score**	**Preoperative**	13.6±1.5	13.6±1.3	0.759
	**Postoperative**	6.5±0.9	10.6±1.1	<0.001*
	**◊p-value**	<0.001*	<0.001*	
**VAS of surgeon**	**Preoperative**	7.9±1.5	8.0±0.8	0.872
	**Postoperative**	1.6±0.5	3.5±0.8	<0.001*
	**◊p-value**	<0.001*	<0.001*	
**Manchester scar plus surgeon's scores**	**Preoperative**	21.5±2.8	21.6±1.7	0.795
	**Postoperative**	8.1±1.1	14.1±1.3	<0.001*
	**◊p-value**	<0.001*	<0.001*	
**Patient satisfaction score by VAS**	**Preoperative**	7.6±1.2	7.7±0.8	0.900
	**Postoperative**	2.6±0.5	5.2±1.2	<0.001*
	**◊p-value**	<0.001*	<0.001*

## Discussion

Linear facial scars have been stigmatizing people in several communities and have become a major psychological issue rather than a physical one. Furthermore, individuals who sustain these accidental injuries are unfairly perceived as being violent individuals. Despite the numerous advancements in the treatment of unsightly scars, especially minimally invasive modalities, not all patients receive complete information regarding the most suitable treatment options for their scars. This has led to the exhaustion of financial and emotional resources in search of solution to this distress. Although complete disappearance of unsightly scars is not guaranteed, a perfect solution can still exist for every scar. With adequate patient education, careful scar assessment and courage, we can eliminate this stigma by creating a camouflaged scar that can blend within the facial features.

Several patients dealing with scars from 2021 to 2023 were treated at our center and we noticed that their first preference was not surgical revision but rather resurfacing by fractionated CO_2_ laser given its high popularity within all current plastic surgery centers and its remarkable benefits. It is an undeniable fact that laser resurfacing is and will remain an important aiding modality in scar improvement as shown in a study that was conducted in 2009.^7^ The author had compared different types of laser modes and mechanisms such as pulsed dye laser (PDL) with fractionated laser with remarkable results in scar appearance in the second group. They stated that minimal complications were noted with noticeable enhancement in pigmentation and scar thickness. Microneedling is among the strongest tools in scar enhancement and is known for its minimally invasive nature, safety and variety of uses especially in atrophic scars.^8^ Although it is appealing and used in our current practice, some studies demonstrated that laser resurfacing is not superior when compared with other techniques in treating scars; therefore, laser resurfacing has been used due to its availability and practicality.^9^ Although it may improve the results, it does not change the orientation of the original scar and patients will always be chained to a vicious cycle of emotional burden. However, as we searched the literature, we did not find a clear comparison between isolated laser resurfacing using fractional CO_2_ laser and surgical revision. Therefore, we decided to compare both techniques in search of a groundbreaking change in scar management.

We used an irregularization technique with our scar patients to deceive the eye into thinking that there was no scar in the first place, which was clearly studied by Borges in 1977, who analyzed scars and scar revision objectives.^10^ The same author published an article in 1984, mainly to study the principles of scar camouflage, he highlighted the importance of redirecting scars to run within the relaxed skin tension lines (RSTLs) of the face.^11^ The author also stressed on its uncountable benefits and that it concealed the scar and had a leveling effect allowing the scar to be aligned with other unscarred areas, thus, achieving homogeneity.^12^ This made techniques such as z-plasty invaluable in counteracting deformities such as trap door scars.^13^, ^14^ Lastly, Borges emphasized about the ability of zigzag scars to stick together during healing, limiting its propensity to widen.^11^

During our study and follow-up, we witnessed these established concepts in action. Among the 122 patients, 59 agreed to have their scars re-oriented with post-operative fractional laser resurfacing sessions. We noticed a prominent enhancement in scar appearance and patients’ satisfaction with a statistically significant improvement in pre- and post-operative Manchester scar scores. A comparison between straight line closure and w-plasty in 2021 demonstrated a pronounced satisfaction among patients who had their scars revised via w-plasty.^15^,^15^ Although this study results matched our results regarding patients’ satisfaction while using a similar concept for camouflage, the authors had based their results solely on subjective VAS. Therefore, we considered using a reliable and objective scale such Manchester scar score (MSS) as a strength point in our study. We also tried to minimize the complications reported such as widening, wound dehiscence, wound infections and haematoma after surgical revision by meticulous asepsis and gentle tissue handling along with minimizing tension during approximation. We noticed minimal surgical site complications that were treated and resolved instantly. Five patients who underwent scar tissue re-arrangement were not compliant to their laser resurfacing schedule; therefore, they were excluded from our study. However, in a yearly follow-up arranged by our practice, the patients’ scores and satisfaction were far less than those in the observed with the combined approach. Thus, the combined approach may potentially offer superior results in linear scar management.

This study had limitations that we hope we overcome in future implementations and further research of this technique's reliability. Our study had a larger male population presenting for both treatments, which may have caused some mismatched results. We also believe that this study would have been stronger if a larger sample size was involved. Finally, a randomized controlled trial would be ideal to standardize this technique and produce the most accurate results.

## Conclusion

Fractional laser resurfacing and scar re-orientation combined with laser treatment are effective in improving the appearance of linear facial scars. However, the combined surgical approach demonstrated improved outcomes in the final appearance, with a positive impact on patients’ quality of life and psychological health.

## Statistical Methods

The collected data were coded, tabulated and statistically analyzed using IBM SPSS statistics (Statistical Package for Social Sciences) software version 28.0, IBM Corp., Chicago, USA, 2021. Quantitative data described as mean±SD (standard deviation), and then compared using the independent t-test and paired t-test. Qualitative data described as number and percentage and then compared using the chi squared, Fisher's exact, McNemar and Marginal homogeneity tests. The level of significance was set at p-value≤0.050, otherwise it was considered non-significant.

## Conflict of Interest

No conflict of interests were present while working on this paper.
